# Topological isoconductance signatures in Majorana nanowires

**DOI:** 10.1038/s41598-021-96415-3

**Published:** 2021-08-27

**Authors:** L. S. Ricco, J. E. Sanches, Y. Marques, M. de Souza, M. S. Figueira, I. A. Shelykh, A. C. Seridonio

**Affiliations:** 1 Science Institute, University of Iceland, Dunhagi-3, 107 Reykjavik, Iceland; 2 School of Engineering, Department of Physics and Chemistry, São Paulo State University (Unesp), 15385-000 Ilha Solteira, SP Brazil; 3 Department of Physics, ITMO University, 197101 St. Petersburg, Russia; 4 Department of Physics, São Paulo State University (Unesp), IGCE, 13506-970 Rio Claro, SP Brazil; 5 Instituto de Física, Universidade Federal Fluminense, 24210-340 Niterói, Rio de Janeiro Brazil

**Keywords:** Condensed-matter physics, Electronic properties and materials, Topological matter

## Abstract

We consider transport properties of a hybrid device composed by a quantum dot placed between normal and superconducting reservoirs, and coupled to a Majorana nanowire: a topological superconducting segment hosting Majorana bound states (MBSs) at the opposite ends. It is demonstrated that if highly nonlocal and nonoverlapping MBSs are formed in the system, the zero-bias Andreev conductance through the dot exhibits characteristic isoconductance profiles with the shape depending on the spin asymmetry of the coupling between the dot and the topological superconductor. Otherwise, for overlapping MBSs with less degree of nonlocality, the conductance is insensitive to the spin polarization and the isoconductance signatures disappear. This allows to propose an alternative experimental protocol for probing the nonlocality of the MBSs in Majorana nanowires.

## Introduction

In last years, the seek for the so-called Majorana bound states (MBSs) has become one of the hottest research fields in the condensed matter physics^1–3^. Besides fundamental interest, the unambiguous experimental detection of these exotic non-Abelian excitations is considered to be the first step towards the realization of a fault-tolerant topologically protected quantum qubit^4–6^. Currently, there exist a plethora of theoretical proposals of the geometries where MBSs can emerge^3^. One of the most promising alternatives is the system consisting of a segment of a quasi-one-dimensional semiconducting nanowire with strong Rashba spin-orbit (SO) coupling, brought in contact with a *s-*wave superconductor and placed into external longitudinal magnetic field.

In this setup, the proximitized nanowire is driven into the regime of unusual *p-*wave superconductivity and thereafter, if the value of the magnetic field exceeds the critical one, reaches the topological phase with nonoverlapping MBSs appearing at the edges^7,8^. The presence of a quantized and robust zero-bias peak (ZBP), which is expected to appear in tunneling spectroscopy probe measurements^9–17^ is considered an indicative of the presence of the isolated MBSs in these so-called Majorana nanowires^16,18,19^. Unfortunately, other mechanisms can be responsible for the appearance of ZBPs, as for instance the formation of zero-energy Andreev bound states (ABSs)^20–29^ and disorder^17,19,27,30,31^. In some cases, these topologically trivial subgap states become pinned at zero-energy for a broad range of system parameters, mimicking exactly the behavior of truly topological MBSs^19,26–28,32–36^. In spite of both recent theoretical and experimental efforts to distinguish the genuine ZBP coming from topologically protected MBSs and the spurious one brought forth by topologically trivial ABSs^13,17,28,35,37–50^, there is still no satisfactory solution of the problem, and the deadlock remains on the table.

For Majorana nanowires with finite-length, one of the key features of MBSs and other trivial subgap zero-energy states lie on the localization of such states in the nanowire ends^12,13,28,34,37–39^. The ideal case corresponds to topological MBSs well-localized at the edges of pristine and long nanowires, with exponentially suppressed overlap between them^3,27,28^. Otherwise, although still within the topological phase, MBSs can overlap with each other when the nanowire is not long enough to ensure the exponential suppression between them, with corresponding MBSs wave functions spread across the device with an exponential decay that oscillates with applied magnetic field and chemical potential, for instance^28,39,51–53^. Moreover, trivial subgap states with apparent nonlocality^34,35,39,46^ may arise in inhomogeneous nanowires^19,20,25,27,30,39,54^, as the so-called partially separated ABS (ps-ABS)^55^ or quasi-MBSs^34^.

Despite the importance of characterizing the topological and trivial nature in this realm of subgap zero (or near-zero) states^13,19,27,28,30,35,38,39,56^, it is also pivotal to estimate “how nonlocal” are these bound states, since the fault-tolerant ability of Majorana-based quantum computing operations strongly relies on its nonlocal feature^4,6,34,39^. In Refs.^38^ and^37^, it was proposed to estimate the degree of Majorana nonlocality in a Majorana nanowire using a quantum dot (QD) as a local probe, which was then experimentally performed in Ref^13^. Theoretically, this nonlocality is estimated by computing the ratio between the couplings of the QD with both the MBSs hosted in the nanowire^38^, which also defines the so-called topological quality factor^37,57^.

**Figure 1:**
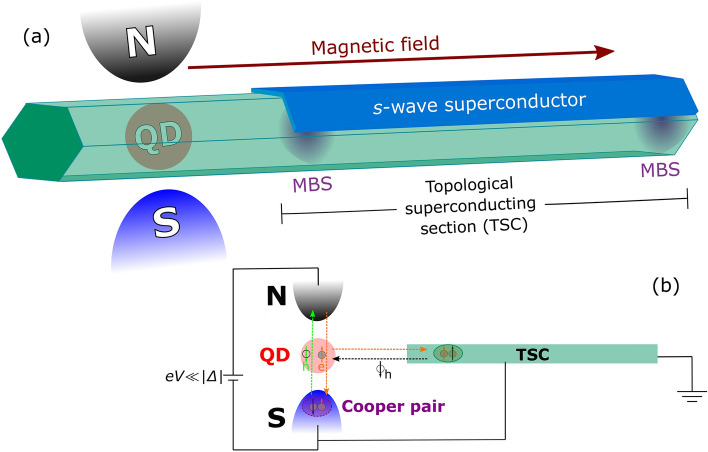
(**a**) Sketch of the considered setup. It consists of a QD working as a local probe coupled to normal (N) and superconducting (S) leads and a segment of a semiconductor nanowire covered by an *s*-wave superconductor layer. In the presence of an external magnetic field parallel to the wire, the latter is driven into a topological superconducting state, with Majoranas bound states (MBSs) formed at its opposite ends. (**b**) The scheme illustrating spin-dependent transport channels in the system. Finite bias voltage *eV* is applied between superconducting (S) and normal (N) reservoirs. An incoming electron from the normal reservoir with a certain spin is injected into the QD and is reflected back as a hole. In the same time, a Cooper pair is formed either in the superconducting reservoir, where it has ordinary *s*-wave character, or in the TSC, where it has a *p*-wave symmetry. The interplay between the transport through S and TSC terminals defines the spin orientation of the reflected hole with respect to the spin of the incoming electron.

In the current work, by analyzing the Andreev current through a quantum dot (QD) placed between metallic (N) and superconducting (S) reservoirs and coupled to a TSC hosting MBSs (Majorana nanowire), see Fig. 1a^58–61^, we theoretically propose an additional protocol to differentiate between the corresponding foregoing cases of:

(A) Highly nonlocal and nonoverlapping MBSs: corresponds to the case of long nanowires, $L\gg \xi _{M}$, wherein *L* is the TSC section length and $\xi _{M}$ is the Majorana coherence length^28^. In such a situation, the wave functions of the MBSs are well localized at the TSC nanowire ends, leading to both a zero overlap between them and a zero coupling of QD state with the faraway (right) MBS.
(B) Nonlocal and overlapping MBSs: describes the opposite case of shorter nanowires ($L\lesssim \xi _{M}$), wherein the wave functions of MBSs can overlap with each other^28,51,53,62^ and the QD also can couple with the outer (right) Majorana state^13,34,37,38^.

For the ideal situation of nonoverlapping and highly nonlocal MBSs (A), the Andreev conductance profiles reveal strong dependence on the parameter which characterizes the spin asymmetry of the coupling between the QD and the TSC. More specifically, the zero-bias Andreev conductance as a function of both the gate-voltage defining the position of the energy level of the QD and the strength of the hybridization between the QD and superconducting lead exhibits isoconductance lines with maximum value of $e^{2}/h$. Their shape strongly depends on the spin asymmetry of the system. However, for the opposite case of overlapping MBSs with lower degrees of nonlocality (B), the sub-gap Andreev conductance becomes spin-independent, and the aforementioned isoconductance profiles with its characteristic $e^{2}/h$ value disappear. Instead, the Andreev conductance at zero-bias shows either a non-quantized peak or a dip, depending on the relative values of parameters which characterizes the direct overlap between the MBSs and the coupling between the QD and the outer (right) MBS. Thus, our findings contribute to the endeavor of characterizing the nonlocality of MBSs by means of sub-gap Andreev conductance measurements using a QD as a local probe.

## Results and discussion

In what follows, we analyze the sub-gap Andreev conductance at zero-temperature limit using the value of $\Gamma _{N}$ as energy unit, with has the order of $\upmu \hbox {eV}$^18,57^ and fixing $\lambda _{L}=2.0\Gamma _{N}$, $U=2.0\Gamma _{N}$ and $V_{Z}=1.2\Gamma _{N}$ [Eq. (4)] for (A) highly nonlocal and nonoverlapping MBSs and (B) nonlocal and overlapping MBSs. The coupling $\lambda _{L}>\Gamma _{N}$ is chosen to ensure that the coupling of the QD with the lead does not spoil any features coming from the QD-TSC hybridization. Moreover, it should be mentioned that for finite temperatures, the sub-gap Andreev resonances are broadened while keeping its area, leading to a reduction of its height^52^. In this scenario, the temperature should be smaller than the resonances width ($k_{\text {B}}T\ll \Gamma _{N}$)^52,63,64^. Otherwise, the corresponding resonances are experimentally invisible. Moreover, the temperature also has a scaling function with the QD-MBSs couplings $\lambda _{(L,R)}$, so that the resonances are visible for $k_{\text {B}}T \ll \lambda _{(L,R)}$^34^.

### Highly nonlocal and nonoverlapping MBSs

**Figure 2:**
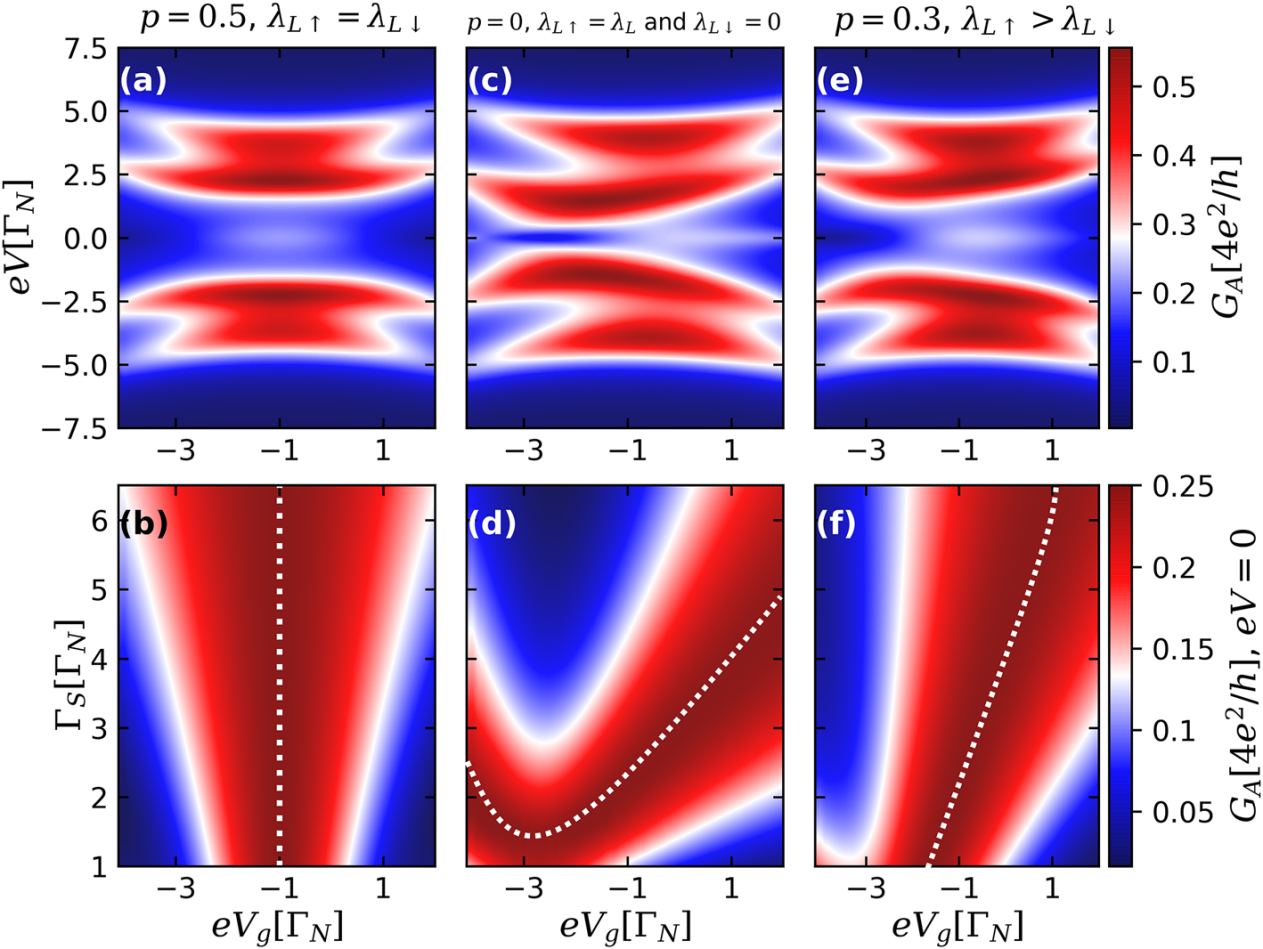
Upper panels: Color scale plots of the Andreev conductance $G_A$ [Eq. (5)] as a function of bias voltage *eV* and QD gate voltage $eV_{g}$, for the case of nonoverlapping ($\varepsilon _{\text {M}}=0$) and highly nonlocal MBSs ($\lambda _{R\sigma }=0$), corresponding to ideal topologically protected situation, with $\Gamma _{S}=3.0\Gamma _{N}$. Panels (**a**,**c**,**e**) correspond to the cases of spin-independent ($p=0.5$, $\lambda _{L\uparrow }=\lambda _{L\downarrow }$), fully spin-polarized ($p=0$, $\lambda _{L\uparrow }=\lambda _{L}$ and $\lambda _{L\downarrow }=0$) and intermediate ($p=0.3$, $\lambda _{L\uparrow }>\lambda _{L\downarrow }$) QD-TSC couplings, respectively. Lower panels: Color scale plots of Andreev conductance at zero-bias as a function of the QD-S hybridization strength $\Gamma _{S}$ and $eV_{g}$ for same values of the parameter *p* as in the upper panels. White dotted lines correspond to *isoconductance* lines, defined by the condition that the conductance reaches its maximal value, $G_A(eV=0)=e^2/h$.

We start with the ideal situation of nonoverlapping ($\varepsilon _{\text {M}}=0$) and highly nonlocal MBS ($\lambda _{R\sigma }=0$), with spin-independent QD-TSC coupling, putting $p=0.5$, $\lambda _{L\uparrow }=\lambda _{L\downarrow }=\lambda _{L}/2$. Figure 2a shows the Andreev conductance as a function of both the bias-voltage *eV* and the gate-voltage $eV_{g}$, which shifts the position of the energy levels of the QD, for $\Gamma _{S}=3.0\Gamma _{N}$. One can clearly notice the presence of the pronounced four peak structure around $eV=0$ corresponding to the well resolved Andreev levels, appearing due to the QD-TSC coupling and splitted by the external magnetic field. Moreover, there is a visible zero-bias structure present because of the leakage of an isolated MBSs into the QD^18,58,61,65^, whose amplitude $G_{A}(eV=0)$ changes with $eV_{g}$, and reaches the maximal value of $e^{2}/h$ for $eV_{g}=-1.0\Gamma _{N}$.

In Fig. 2b we demonstrate how Andreev conductance amplitude at zero-bias also changes as a function of both $eV_{g}$ and QD-S hybridization strength $\Gamma _{S}$ for the same case of $p=0.5$. Experimentally, one can change $\Gamma _{S}$ continuously while tuning the QD level $eV_{g}$ by employing a dual-gate device geometry^66^. The maximal value of the conductance $e^{2}/h$ is reached along the white vertical dotted line, which we call *isoconductance* line. For this particular spin-independent situation, the position of this line is defined by the condition of particle-hole symmetry, reached when $eV_{g}=-1.0\Gamma _{N}$. This condition is broken in spin asymmetric case, when $\lambda _{\uparrow }\ne \lambda _{\downarrow }$^60^, which leads to the distortion of the isoconductance line in the $(eV_{g},\Gamma _{S})$ space, as we shall see. Note also that along the isoconductance line, the zero bias conductance does not depend on the value of $\Gamma _{S}$, so the QD becomes effectively decoupled from the S lead and the transport through it is uniquely defined by its pairing to the TSC.

**Figure 3:**
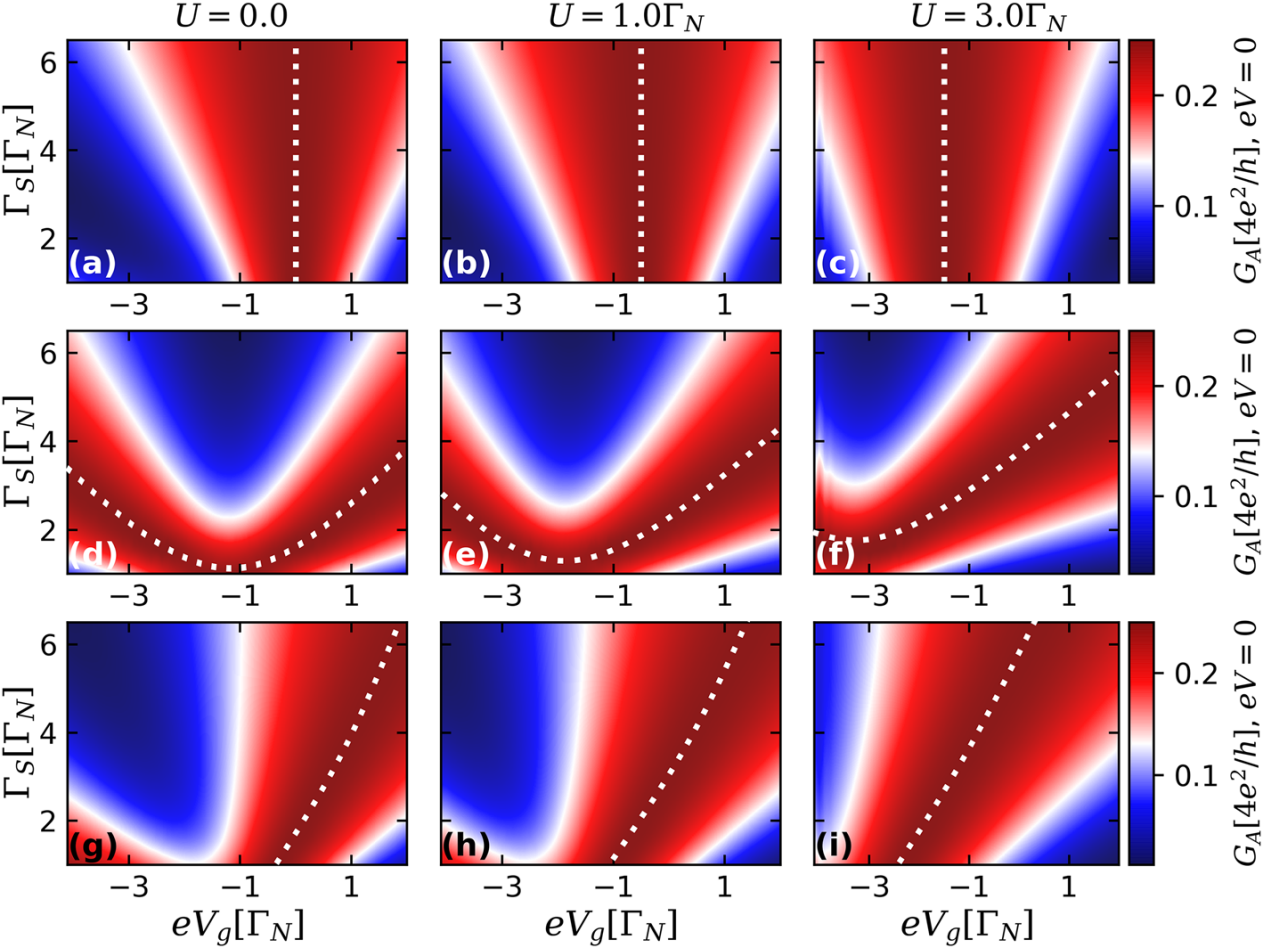
Color scale plots of Andreev conductance [Eq. (5)] at zero-bias as a function of the QD-S hybridization strength $\Gamma _{S}$ and the QD gate-voltage $eV_{g}$ for the case of nonoverlapping ($\varepsilon _{\text {M}}=0$) and highly nonlocal MBSs ($\lambda _{R\sigma }=0$), corresponding to ideal topologically protected situation, considering several values of Coulomb correlation strength *U*. Upper panels correspond to the spin-independent QD-TSC coupling ($p=0.5$, $\lambda _{L\uparrow }=\lambda _{L\downarrow }$), while middle and lower panels depict the fully-spin polarized ($p=0$, $\lambda _{L\uparrow }=\lambda _{L}$ and $\lambda _{L\downarrow }=0$) and the case of intermediate polarization ($p=0.3$, $\lambda _{L\uparrow }>\lambda _{L\downarrow }$), respectively. White dotted lines correspond to *isoconductance* lines, defined by the condition that the conductance reaches its maximal value, $G_A(eV=0)=e^2/h$.

**Figure 4:**
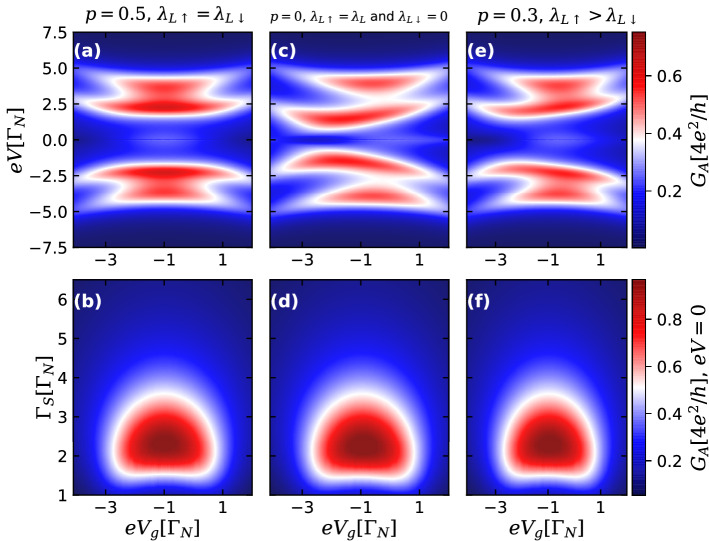
Upper panels: Color scale plots of the Andreev conductance $G_A$ [Eq. (5)] as a function of bias voltage *eV* and QD gate voltage $eV_{g}$, for the case of overlapping MBSs well-localized at edges of the TSC section ($\varepsilon _{\text {M}}=0.05\Gamma _{N}$,$\lambda _{R\sigma }=0$) and $\Gamma _{S}=3.0\Gamma _{N}$. Panels (**a**), (**c**) and (**e**) correspond to the cases of spin-independent ($p=0.5$, $\lambda _{L\uparrow }=\lambda _{L\downarrow }$), fully spin-polarized ($p=0$, $\lambda _{L\uparrow }=\lambda _{L}$ and $\lambda _{L\downarrow }=0$) and intermediate ($p=0.3$, $\lambda _{L\uparrow }>\lambda _{L\downarrow }$) QD-TSC couplings, respectively. Lower panels: Color scale plots of Andreev conductance at zero-bias as a function of the QD-S hybridization strength $\Gamma _{S}$ and $eV_{g}$ for same values of parameter *p* as in the upper panels. Note, that differently from the case of isolated MBSs illustrated by Fig.2, the value of the zero bias conductance $G_A(eV=0)$ can exceed $e^2/h$, and the *isoconductance* lines are absent.

**Figure 5:**
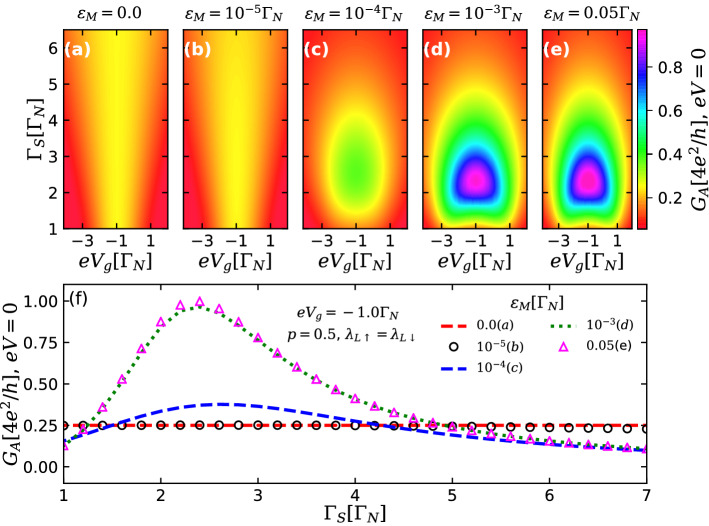
(**a**–**e**) Color scale plots of the Andreev conductance $G_A$ [Eq. (5)] at zero-bias as a function of the QD-S hybridization strength $\Gamma _{S}$ and QD gate-voltage $eV_{g}$ for spin symmetric case ($p=0.5$), for five distinct values of the parameter $\varepsilon _{\text {M}}$ defining the overlap between the MBSs well-localized at the ends of the TSC section ($\lambda _{R\sigma }=0$). One clearly sees that condition $G_A(eV=0)=e^2/h$ is satisfied along the open vertical line (*isoconductance* line) in the left two panels corresponding to highly isoladed MBSs, and along the closed line in the right three panels, corresponding to highly overlapping MBSs. In this latter case, the value of the conductance inside the line exceeds $e^2/h$ (**f**): Andreev conductance at zero-bias plotted as a function of $\Gamma _S$ with $eV_{g}=-1.0 \Gamma _{N}$, for the same values of $\varepsilon _{\text {M}}$ as in the upper panels.

**Figure 6:**
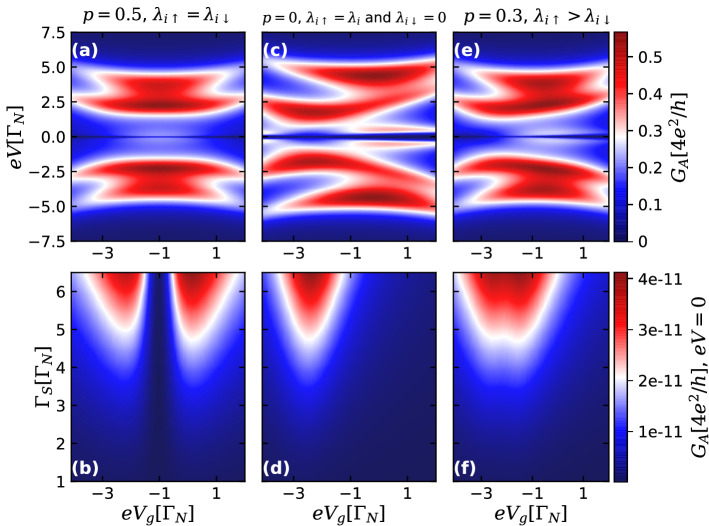
Upper panels: Color scale plots of the Andreev conductance $G_A$ [Eq. (5)] as a function of bias voltage *eV* and QD gate voltage $eV_{g}$, for the case of nonlocal MBSs ($\lambda _{R}=0.5\Gamma _{N},\lambda _{L}=2.0\Gamma _{N}$) with small overlap ($\varepsilon _{M}\ll \lambda _{R}$) and $\Gamma _{S}=3.0\Gamma _{N}$. Panels (**a**), (**c**) and (**e**) correspond to the cases of spin-independent ($p=0.5$, $\lambda _{i\uparrow }=\lambda _{i\downarrow }$), fully spin-polarized ($p=0$, $\lambda _{i\uparrow }=\lambda _{i}$ and $\lambda _{i\downarrow }=0$) and intermediate ($p=0.3$, $\lambda _{i\uparrow }>\lambda _{i\downarrow }$) QD-TSC couplings, respectively, with $i=L,R$. Lower panels: Color scale plots of Andreev conductance at zero-bias as a function of the QD-S hybridization strength $\Gamma _{S}$ and $eV_{g}$ for same values of *p* as in the upper panels. Note that differently from the cases of nonoverlapping (Fig.2) and overlapping (Fig.4) MBSs well-localized at the edges of the TSC section, the value of the zero-bias conductance $G_A(eV=0)$ drops to zero, and the *isoconductance* lines are absent.

**Figure 7:**
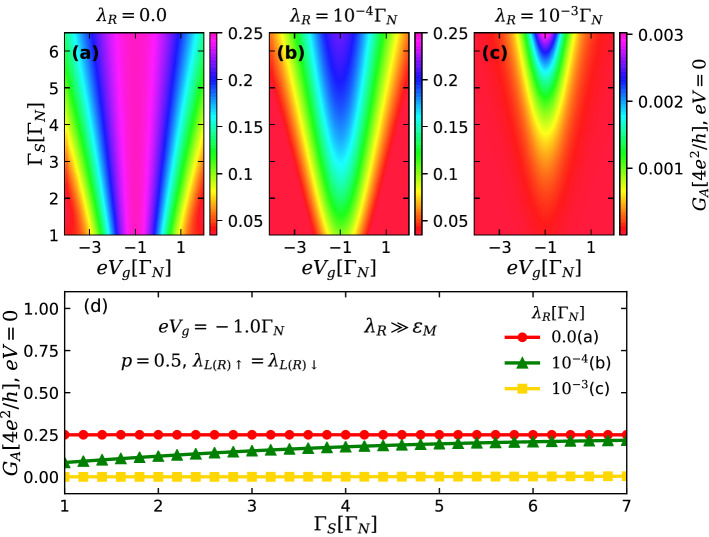
(**a**–**c**) Color scale plots of the Andreev conductance $G_A$ [Eq. (5)] at zero-bias as a function of the QD-S hybridization strength $\Gamma _{S}$ and QD gate-voltage $eV_{g}$ for spin symmetric case ($p=0.5$) and $\lambda _{L}=2.0\Gamma _{N}$, considering three distinct values of the parameter the QD-right MBS coupling $\lambda _{R}$ defining the nonlocality of the MBSs for $\lambda _{R}\gg \varepsilon _{M}$. One can easily verify an *isconductance* line with $G_{A}(eV=0)=e^{2}/h$ in panel (**a**) for the case of highly nonlocal Majoranas ($\lambda _{R}=0$). However, the *isoconductance* profile is completely destroyed even for small values of $\lambda _{R}$ and approaches to zero, as shown in panels (**b**) and (**c**), respectively. (**d**) Andreev conductance at zero-bias plotted as a function of $\Gamma _{S}$ with $eV_{g}=-1.0\Gamma _{N}$, for the same values of $\lambda _{R}$ as in the upper panels.

**Figure 8:**
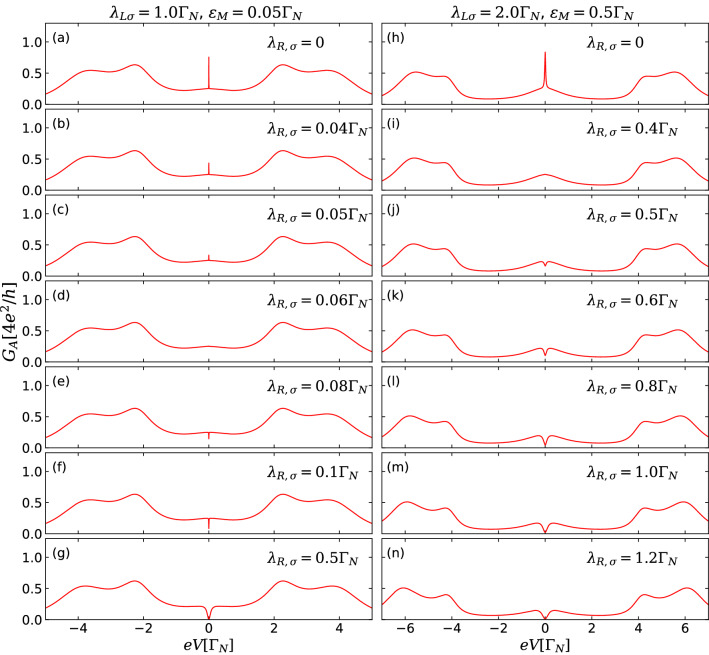
Andreev conductance $G_A$ [Eq. (5)] as a function of the bias-voltage for spin symmetric case ($p=0.5$) and distinct values of $\lambda _{R\sigma }$. In left panels (**a**–**g**), $\varepsilon _{M}=0.05\Gamma _{N}$ and the other parameters are the same adopted in Fig. 7: $\lambda _{L\uparrow }=\lambda _{L\downarrow }=1.0\Gamma _{N}$, $eV_{g}=-1.0\Gamma _{N}$, $\Gamma _{S}=3.0\Gamma _{N}$ and $V_{Z}=1.2\Gamma _{N}$. In right panels [(h)-(n)], $\varepsilon _{M}=0.5\Gamma _{N}$ and we adopt $\lambda _{L\uparrow }=\lambda _{L\downarrow }=2.0\Gamma _{N}$, $eV_{g}=-1.5\Gamma _{N}$, $\Gamma _{S}=4.0\Gamma _{N}$ and $V_{Z}=2.0\Gamma _{N}$. In both the set of parameters chosen the Anderson symmetric condition $U= 2|eV_{g}|$ is fulfilled.

**Figure 9:**
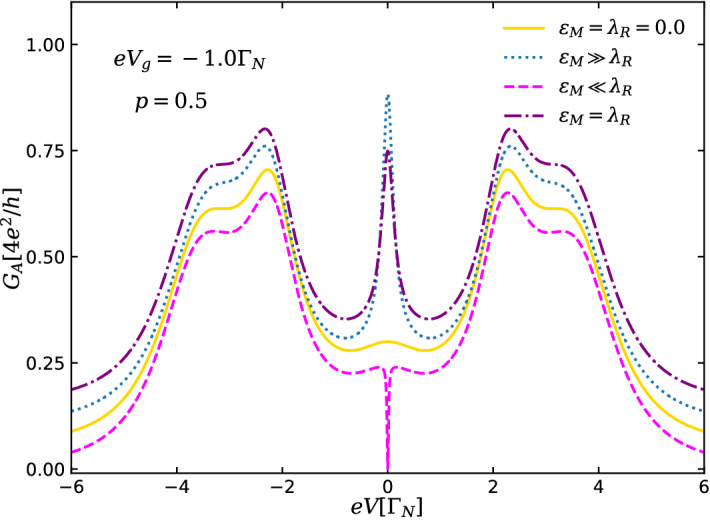
Andreev conductance $G_A$ [Eq. (5)] as a function of the bias-voltage for spin symmetric case ($p=0.5$), with $\lambda _{L}=2.0\Gamma _{N}$, $\Gamma _{S}=3.0\Gamma _{N}$ and $eV_{g}=-1.0\Gamma _{N}$. The lines are vertically offset for a better viewing. Solid orange line corresponds to ideal case of highly nonlocal and nonoverlapping MBSs ($\varepsilon _{M}=\lambda _{R}=0$), while teal dotted and magenta dashed lines describe the situations of overlapping but well-localized Majoranas ($\varepsilon _{M}\gg \lambda _{R}$) and quasi-MBSs ($\lambda _{R}\gg \varepsilon _{M}$), respectively. The case where the overlap between the MBSs and the QD-right MBS are on equal foot ($\varepsilon _{M}=\lambda _{R}$) is described by the purple dash-dotted line.

The opposite case of fully spin polarized transport, corresponding to $p=0$, $\lambda _{\uparrow }=\lambda$ and $\lambda _{\downarrow }=0$ is illustrated by Fig. 2c,d. The profile of the conductance as a function of the bias and gate-voltages becomes asymmetric, as it can be clearly seen in Fig. 2c. Zero-bias conductance peak still appears, but the isoconductance line defined by the condition $G_A(eV=0)=e^2/h$ is not a straight vertical line, but has a more complicated shape shown in Fig. 2d. Note that differently from the case shown in Fig. 2b, the isoconductance line has a minimum, which means that maximal value of the zero-bias conductance $e^2/h$ can not be reached below certain critical value of the coupling between the QD and the S lead. The intermediate case of $p=0.3$ is illustrated by Fig. 2e,f.

The comparison between the three sets of panels of Fig. 2 allows us to conclude that the presence of an isoconductance plateau corresponding to the vertical isoconductance line in $eV_g,\Gamma _S$ coordinates can be considered as a hallmark of spin symmetric coupling between the QD and the TSC.

It is worth mentioning that $e^{2}/h$ quantized Andreev conductance amplitude characteristic for highly nonlocal and nonoverlapping MBSs ($\varepsilon _{M}=\lambda _{R\sigma }=0$)^60^ at $T=0$ is distinct from $2e^{2}/h$ value typical for the normal conductance through a N-QD-N geometry^67^ without the presence of the TSC section ($\lambda _{L\sigma }=0$). In such case, away from the Kondo regime, for *e.g.* spin-polarized coupling ($\lambda _{L\uparrow }\ne 0$ and $\lambda _{L\downarrow }=0$), the density of states corresponding to the spin $\uparrow$ drops to $e^{2}/2h$ owing to the coupling with an isolated MBS, while the same quantity for the spin $\downarrow$ remains unaffected ($e^2/h$), giving rise to a ZBP height of $3e^{2}/2h$^67,68^. However, if a N-QD-S geometry is considered, the pairing induced into the QD by the S lead mixes the spins [Eq. (4)] and hence both spin channels are affected by the presence of the MBSs, even in the fully spin-polarized situation with $p=0$^60^. This interference process between the spin channels mediated by the S-lead reduces the Andreev conductance maximum amplitude from $4e^{2}/h$ to $e^{2}/h$ (see *e.g.* Fig. 5).

Although finite temperature effects can flatten the isoconductance plateaus of Fig. 2^64^, quantized conductance values near $e^{2}/h$ may still be obtained in a realistic experimental situation when the QD-TSC coupling $\lambda _{L\sigma }$ is dominant over both the temperature $k_{B}T$ and the overlap $\varepsilon _{M}$ between the MBSs^41,69^. Previously some of us have studied the interplay between the thermal broadening $k_{B}T$ and the overlap strength $\varepsilon _{M}$ of the MBSs via NRG analysis^69^. It was shown that overlapping MBSs can become decoupled from each other when the system is driven into a specific fixed point due to finite temperature effects, fully recovering the ZBP signature.

In Fig. 3, we analyze the behavior of the isoconductance profiles with the Coulomb correlation *U* for the ideal case of nonoverlapping ($\varepsilon _{M}=0$) and highly nonlocal MBSs ($\lambda _{R\sigma }=0$), for the same values of spin anisotropy parameter *p* as in Fig. 2. For the spin-independent case (Fig. 3a–c), the isoconductance lines depicted by the white-dashed lines have their positions at the $eV_{g}$-axis shifted as *U* changes, being pinned at $eV_{g}=-U/2$. The corresponding fully-spin polarized and partially spin-polarized cases are illustrated by the middle [(d–f)] and lower panels [(g–i)] of Fig. 3, respectively. They also reveal similar shift with the change of *U*, demonstrating the robustness of isoconductance with respect to electron-electron interactions.

It should be noted that introduction of the additional transport channels (multiple subbands) in the TSC nanowire of a considerable thickness can lead to the vanishing of isoconductance signatures of highly isolated MBSs^28,70,71^, as the nonzero occupancy of multiple subbands plays role of an effective disorder^71^, thus leading to the formation of trivial low-energy states which may emulate the signatures of MBSs. One thus needs to use narrow wires with low values of chemical potential, with only few occupied subbands^71^, which is fully experimentally feasible.

### Nonlocal and overlapping MBSs

Now, let us analyze the case of MBSs at opposite edges of shorter nanowires ($L\lesssim \xi _{M}$), leading to a finite overlap between them ($\varepsilon _{\text {M}}=0.05\Gamma _{N}$) and considering first $\lambda _{R}=0$. Figure 4 illustrates the cases of spin-independent ($p=0.5$), fully spin-polarized ($p=0$) and intermediary ($p=0.3$) QD-TSC couplings. The assumption of this finite overlap leads to the formation of a nonlocal fermionic state with energy $\varepsilon _{M}$ coming from the combination of the Majorana states at opposite ends of the TSC. This can be verified by rewriting the Majorana operators of Eq. (2) as a combination of standard Dirac operators for electrons and holes^3,4^.

In the upper panels, Andreev conductance as a function of the bias and gate-voltages for the fixed value of $\Gamma _S=3.0\Gamma _N$ is shown. Direct comparison with upper panels of Fig. 2 shows that conductance profiles are qualitatively the same for the cases of topological nonoverlapping MBSs. However, if one turns to zero-bias conductance as a function of the gate voltage $eV_g$ and QD-S lead coupling $\Gamma _S$, the results are totally different. It was already stated that for the case of highly nonlocal and nonoverlapping MBS ($\varepsilon _{M}=\lambda _{R\sigma }=0$), the maximal value $G_A(eV=0)=e^2/h$ is reached along certain open isoconductance lines (Fig. 2b,d,f). The situation for the case of overlapping MBSs is qualitatively different. Indeed, it can be clearly seen from the lower panels of Fig. 4 that the condition $G_A(eV=0)=e^2/h$ at zero-temperature is reached along the closed lines, which now can not be considered as isoconductance lines, as inside them the value of the conductance exceeds $e^2/h$. This remarkable difference suggests a further experimental criterion for distinguishing between the topological cases^28,38^ of overlapping and nonoverlapping MBSs localized at the edges of the TSC nanowire.

To study in more detail the corresponding crossover, we analyzed the zero-bias Andreev conductance as a function of $eV_g$ and $\Gamma _S$ for several values of the parameter $\varepsilon _{M}$, characterizing the overlap between the different MBSs well-localized at the edges. The results are shown in Fig. 5. In the panels (a–e) one can clearly see how an open isoconductance line corresponding to the maximal conductance value $G_A(eV=0)=e^2/h$, observable for small $\varepsilon _M$, changes into a closed contour within which the conductance peak exceeding the value of $e^2/h$ raises. The dependence of the maximal conductance on $\Gamma _S$ for the fixed value of $eV_g$ is shown in the panel (f). The conductance plateau characteristic for topological nonoverlapping and nonlocal MBSs is slightly modified for small values of $\varepsilon _M$, corresponding to the situation of almost-zero-energy MBSs, or equivalently, a nonlocal fermionic state with energy level $\varepsilon _{M}$ near zero (see black open dot and blue dashed lines). Under the increase of $\varepsilon _M$, these quasi-plateaus transform into non-monotonous curves corresponding to the onset of strongly overlapped MBSs, characterizing a nonlocal fermionic state with finite energy.

In Fig. 6 we study the case in which the wave function of right MBSs overlaps with the QD state, leading to a finite coupling between them ($\lambda _{R}\gg \varepsilon _{M}$)^13,37,38^. Within this case, we also consider the situations of spin-independent ($p=0.5$), fully spin-polarized ($p=0$) and intermediary ($p=0.3$) QD-TSC couplings. In the upper panels, Andreev conductance profiles as a function of bias and gate-voltage for $\Gamma _{S}=3.0\Gamma _{N}$, $\lambda _{L}=2.0\Gamma _{N}$ and $\lambda _{R}=0.5\Gamma _{N}$ are shown. One can clearly see, that for all the values of *p* the zero-bias Andreev conductance almost drops to zero, which is quite distinct from the cases of highly nonlocal and nonoverlapping MBSs and overlapping MBSs with $\lambda _{R}=0$, see Figs. 2 and 4 . This pronounced drop is also seen in the lower panels showing $G_{A}(eV=0)$ as a function of the QD gate-voltage $eV_{g}$ and QD-S lead coupling $\Gamma _{S}$. Isoconductance signatures are completely absent for all values of the parameter *p*.

Figure 7a–c shows Andreev conductance profiles as a function of $eV_{g}$ and $\Gamma _{S}$ for increasing values of $\lambda _{R}$, allowing to investigate the crossover from highly nonlocal MBSs ($\lambda _{R}=\varepsilon _{M}=0$) to MBSs with lesser degree of nonlocality ($\lambda _{R}\gg \varepsilon _{M}$). In panel (a), one can easily spot the isoconductance line with $G_{A}(eV=0)=e^{2}/h$ for the case of highly nonlocal Majoranas. However, as its nonlocal feature is suppressed with increase of $\lambda _{R}$, the isoconductance profile disappears and $G_{A}$ approaches to zero, see panels (b) and (c), which is quite distinct from the previous situation of overlapping MBSs well-localized at the TSC section ends ($\varepsilon _{M}\ne 0$, $\lambda _{R}=0$) (Fig. 5), where the zero-bias conductance almost reaches its maximal value of $4e^{2}/h$. The zero-bias Andreev conductance behavior as a function of $\Gamma _{S}$ for $eV_{g}=-1.0\Gamma _{N}$ is shown in panel (d), where the plateau of $e^{2}/h$ appears only for the topologically protected case of highly nonlocal MBSs, corresponding to the flat red dotted line.

The characteristic drop in the Andreev conductance at $eV=0$ shown in Figs. 6 and 7 for $\lambda _{R}\gg \varepsilon _{M}$ comes from interference phenomena between distinct transport channels due to the leakage of the left and right MBSs with different strengths ($\lambda _{L}>\lambda _{R}$). In other words, there is a formation of a nonlocal fermion through the QD coming from the unbalanced combination of left and right MBSs, leading to the above mentionated interference process. This underlying mechanism is quite distinct from that one for the opposite case of $\varepsilon _{M}\gg \lambda _{R}$ (Figs. 4 and 5), where the left and right MBSs localized at opposite ends of the TSC section overlap with each other directly. Hence, the QD perceives the TSC section as a nonlocal fermionic state with energy $\varepsilon _{M}$, giving rise to a peak at $eV=0$ in the Andreev conductance.

In Fig. 8, we investigate the crossover between the opposite cases of $\varepsilon _M\gg \lambda _{R}$ and $\varepsilon _M\ll \lambda _{R}$ for $p=0.5$, considering two distinct sets of parameters corresponding to left (a–g) and right panels (h–n). In the left panels of Fig. 8, the parameters are the same adopted in Fig. 7, but for the specific situation of finite overlap $\varepsilon _{M}=0.05\Gamma _{N}$ between the MBSs. Panel (a) exhibits the situation where the right MBS does not overlap with the QD ($\lambda _{R\sigma }=0$), depicting an Andreev conductance peak at zero-bias with its amplitude higher than the corresponding isoconductance plateau of $e^{2}/h$. This peak at $eV=0$ indicates the formation of a nonlocal fermionic state with energy $\varepsilon _{M}$ coming from the combination between the Majorana components at the opposite ends of the TSC section, as discussed earlier (Fig. 5). However, as the wave function of the right MBS overlaps with QD, the coupling $\lambda _{R\sigma }$ acquire finite values and the Andreev conductance peak at $eV=0$ is suppressed (Fig. 8b–d). Such a conductance drop gets more pronounced when the regime of $\lambda _{R}>\varepsilon _{M}$ is reached (Fig. 8e), leading to the formation of a dip in which the Andreev conductance is strongly suppressed at zero-bias for the situation of $\lambda _{R}\gg \varepsilon _{M}$ (Fig. 8f,g). Similar behavior for this peak-dip transition is also found for other parameters adopted, as seen in the right panels of Fig. 8.

The Andreev conductance profiles shown in Fig. 8 reveal that the peak-dip crossover mechanism at zero-bias is ruled by the relative values of two energy scales: the overlap $\varepsilon _{M}$ between the MBSs and the QD-right MBS hybridization $\lambda _{R}$. It was previously shown that these quantities also govern the emergence of distinct profiles for the QD-MBSs energy spectrum in absence of the S-lead^13,37,38,57^.

In Fig. 9, we summarize the main differences between (A) highly nonlocal and nonoverlapping MBSs and (B) nonlocal and overlapping MBSs in the Andreev conductance spectra as a function of bias-voltage for the spin symmetric case ($p=0.5$), with $\lambda _{L}=2.0\Gamma _{N}$, $\Gamma _{S}=3.0\Gamma _{N}$ and $eV_{g}=-1.0\Gamma _{N}$. For the ideal situation of highly nonlocal and nonoverlapping MBSs ($\varepsilon _{M}=\lambda _{R}=0$, orange solid line), corresponding to Fig. 2a, a ZBP with quantized amplitude of $e^{2}/h$ and satellite peaks describing the Andreev levels formed in the QD due to the coupling with S-lead are observed. For well-localized, but overlapping MBSs ($\varepsilon _{M}\gg \lambda _{R}$, teal dotted line), corresponding to Fig. 4a, the ZBP is not quantized anymore and its height depends on the parameters of the system, as *e.g.* $\Gamma _{S}$ (see Fig. 5). A non-quantized ZBP also characterizes the situation where the overlap between the MBSs is comparable with the QD-right MBS coupling ($\varepsilon _{M}=\lambda _{R}$, purple dash-dotted line). However, for the case where the right MBS wavefunction strongly overlaps with the QD ($\varepsilon _{M}\ll \lambda _{R}$, magenta dotted line) corresponding to Fig. 6a, the ZBP is replaced by a zero-bias dip, which reaches zero (Fig. 7). This peak-dip transition suggests an additional protocol for distinguish between overlapping but well-localized MBSs and less nonlocal MBSs.

Concerning the zero-bias conductance profiles as a function of both the QD-S hybridization $\Gamma _{S}$ strength and the QD gate-voltage $eV_{g}$, we notice that isoconductance lines appear only for the ideal case of highly nonlocal zero-energy MBSs ($\lambda _{R\sigma }=0$) and zero overlap $\varepsilon _{M}$ between each other (Fig. 2, lower panels). For the situation of almost zero-energy MBSs characterized by finite but small $\varepsilon _M$, the plateau which originates the isoconductance lines is slightly distorted (Fig. 5f). Thus, within our effective model we can infer that the robustness of the isoconductance signatures arise from both the zero-energy and nonlocal nature of the MBSs, since for both overlapping MBSs with more ($\varepsilon _{M}\gg \lambda _{R\sigma }$) or less ($\varepsilon _{M}\ll \lambda _{R\sigma }$) nonlocal feature, the MBSs cannot be characterized as true zero-energy and highly nonlocal states anymore. However, since fine-tuned ABSs, quasi-MBSs or disordered-induced zero-energy modes can also induce ZBPs^19,28,30^, it should be emphasized that a study using more detailed models^27,39^ is required in order to investigate if the isoconductance lines can also appear for these topologically trivial subgap states.

## Conclusions

We have studied the sub-gap Andreev conductance $G_A$ through a quantum dot (QD) connected to metallic and superconducting leads and additionally coupled to a hybrid topological semiconducting nanowire (TSC) hosting Majorana bound-states (MBSs) at the opposite ends. For nonoverlapping and highly nonlocal MBSs, corresponding to the ideal case of long and pristine Majorana nanowires, the profiles of $G_A$ as functions of both quantum dot gate-voltage and hybridization between the dot and the superconducting reservoir reveal pronounced isoconductance signatures with maximum amplitude of $e^{2}/h$, sensitive to spin anisotropy of the coupling between the QD and the TSC. However, in the situation of shorter Majorana nanowires, the MBSs remain nonlocal but overlap with each other or lose its nonlocal feature. Hence, such isoconductance signatures disappear, giving rise to a nonquantized zero-bias peak for the former situation and a zero-bias dip for the latter. This suggests that the analysis of the sub-gap Andreev conductance profiles by means of a local probe can be employed as an additional tool to distinguish MBSs with distinct degrees of overlap and nonlocality.

## Methods

### Theoretical model

To describe transport properties of the system sketched in Fig. 1, we use the following Anderson-type Hamiltonian^58,60,72^:

1 $$\begin{aligned} H = \sum _{\alpha =N,S} (H_{\alpha } + H_{\alpha -QD}) + H_{QD} + H_{TSC}, \end{aligned}$$

where $H_{N}=\sum _{{\varvec{k}}\sigma }\varepsilon _{{\varvec{k}}}^{N}c_{N{\varvec{k}}\sigma }^{\dagger }c_{N{\varvec{k}}\sigma }$ and $H_{S}=\sum _{{\varvec{k}}\sigma }\varepsilon _{{\varvec{k}}}^{S}c_{S{\varvec{k}}\sigma }^{\dagger }c_{S{\varvec{k}}\sigma }-\sum _{{\varvec{k}}}(\Delta c_{S{\varvec{k}}\uparrow }^{\dagger }c_{S-{\varvec{k}}\downarrow }^{\dagger } + \text {h.c.})$ represent the N and S reservoirs, respectively, with electron energies $\varepsilon _{{\varvec{k}}}^{\alpha }$, spin $\sigma =\uparrow ,\downarrow$ and superconducting energy gap $\Delta$. $H_{\alpha -QD}=\sum _{{\varvec{k}}\sigma }V_{\alpha {\varvec{k}}\sigma }(c_{\alpha {\varvec{k}} \sigma }^{\dagger }d_{\sigma } + \text {h.c.})$ stands for the hybridization between N(S) reservoir and the QD, characterized by the coupling strength $V_{\alpha {\varvec{k}}\sigma }$. The QD is described by the Hamiltonian $H_{QD} = \sum _{\sigma }\varepsilon _{d\sigma }d_{\sigma }^{\dagger }d_{\sigma }+Un_{d\uparrow }n_{d\downarrow }$, corresponding to a pair of nondegenerate energy levels with the energies $\varepsilon _{d\sigma }=eV_{g}-\sigma V_{Z}$, that can be tuned by a tunnel gate $eV_{g}$ in presence of an external magnetic field inducing the Zeeman splitting $V_{Z}$, and *U* corresponds to the Coulomb repulsion between electrons with opposite spins.

The TSC section can be modeled by the following low-energy effective Hamiltonian^38,73^:

2 $$\begin{aligned} H_{TSC} & =  {} \imath \varepsilon _{M}\gamma _{L}\gamma _{R}+\sum _{\sigma }\left( \lambda _{L\sigma }d_{\sigma }-\lambda _{L\sigma }^{*}d_{\sigma }^{\dagger }\right) \gamma _{L}\nonumber \\&\quad + \sum _{\sigma }\left( \lambda _{R\sigma }d_{\sigma }+\lambda _{R\sigma }^{*}d_{\sigma }^{\dagger }\right) \gamma _{R}, \end{aligned}$$

where the Hermitian operators $\gamma _{i}=\gamma _{i}^{\dagger }(i=L,R)$ describe the MBSs localized at the left (L) and right (R) of the TSC segment [marked in purple in Fig. 1a]^2,3^. The parameter $\varepsilon _{M}$ describes the overlap between the opposite MBSs, while $\lambda _{i,\sigma }(i=L,R)$ characterizes the coupling between the QD and the left/right MBSs, with spin $\sigma =\uparrow ,\downarrow$. The overlap $\varepsilon _{M}$ decays exponentially with the TSC length and oscillates around zero with some system parameters, as the TSC length, chemical potential and applied magnetic field^38,51–53,74^. Hence, $\varepsilon _{M}$ can reach zero at specific values of parameters space (oscillation parity crossings) for shorter TSC sections.

In the highly nonlocal and nonoverlapping case (A), the MBSs are well-localized at the ends of the TSC section ($\lambda _{R}=0$) with an exponentially suppressed overlap between them ($\varepsilon _{M}=0$). However, for the situation of nonlocal and overlapping MBSs (B), the overlap $\varepsilon _{M}$ can be either finite or zero owing to its oscillatory behavior. When considered, $\lambda _{R}$ also can oscillate, but remains finite^38^. For all situations $\lambda _{R}<\lambda _{L}$, once the left MBSs couples with the QD more strongly.

The effective Hamiltonian of Eq. (2) was derived from its corresponding tight-binding model in Ref.^38^. It should be emphasized that the effective model of Eq. (2) trustingly describes the low-energy spectrum of the QD-TSC system^37,38,40^, being able to reproduce qualitatively the experimental results^12,13^. Eq. (2) can be rewritten in the regular spinless fermionic basis by using the transformation $\gamma _{L}=\frac{1}{\sqrt{2}}(f + f^{\dagger })$ and $\gamma _{R}=\frac{\imath }{\sqrt{2}}(f^{\dagger }-f)$^3,75^, with $f^{\dagger }(f)$ being nonlocal fermions with ordinary Fermi-Dirac statistics.

It should be specifically stressed that although the TSC section hosting MBSs is effectively spinless^4,37,53,76^, the coupling of the QD to the MBSs depends on the spin texture of the latter, by means of the canting angles $\theta _{L,R}$^38^ of the left and right MBSs, with $\lambda _{L\sigma }=\lambda _{L}(\sin {\frac{\theta _{L}}{2}},-\cos {\frac{\theta _{L}}{2}})$ and $\lambda _{R\sigma }=-\imath \lambda _{R}(\sin {\frac{\theta _{R}}{2}},\cos {\frac{\theta _{R}}{2}})$, where $\lambda _{i\sigma }\equiv (\lambda _{i\uparrow },\lambda _{i\downarrow })$. These canting angles depend on the applied magnetic field and spin-orbit coupling in the nanowire. These couplings also depend on the effective distance between the QD and the TSC segment^73^. A detailed analysis of the effects of canting angles in the QD-TSC spectrum is beyond our proposal. Thus, the spin-dependency in the QD-MBSs couplings is here accounted by the straightforward introduction of a generic polarization parameter $p \in [0,1]$^60,77^ only for ensuring the possibility of spin asymmetry in such couplings, so that $\lambda _{i,\uparrow }=\lambda _{i}(1-p)$ and $\lambda _{i,\downarrow }=\lambda _{i}p$, where $\lambda _{i}\equiv |\lambda _{i}|$ stands for the maximum coupling amplitudes.

Since we are interested in sub-gap Andreev transport features through the QD and its relation with the MBSs, we restrict ourselves to the limiting case of large superconducting gap $|\Delta |\rightarrow \infty$^60,65,78^. It is well known that in this regime the S lead induces static *s*-wave pairing in the QD due to proximity effect. This allows to trace out the S lead from the Hamiltonian by using the substitution $H_{S} + H_{S-QD}\approx -\Gamma _{S}(d_{\uparrow }^{\dagger }d_{\downarrow }^{\dagger } + \text {h.c.})$^79–82^, where $\Gamma _{S}=\pi \sum _{{\varvec{k}}}|V_{S {\varvec{k}} \sigma }|^{2}\delta (\omega - \varepsilon _{{\varvec{k}}}^{\alpha })$.

Away from the Kondo regime^38,58,66,83^, the effects of the Coulomb blockade in the energy spectrum of the QD coupled to both S and N leads are well-described within the following self-consistent Hartree-Fock approximation (HFA)^38,84–86^:

3 $$\begin{aligned} Un_{d\downarrow }n_{d\uparrow }\approx U(\langle n_{d\downarrow }\rangle n_{d\uparrow }+n_{d\downarrow }\langle n_{d\uparrow }\rangle - \nonumber \\ \langle d_{\downarrow }d_{\uparrow }\rangle d_{\uparrow }^{\dagger }d_{\downarrow }^{\dagger } -\langle d_{\uparrow }^{\dagger }d_{\downarrow }^{\dagger }\rangle d_{\downarrow }d_{\uparrow }) + \text {const.}, \end{aligned}$$

where $\langle n{}_{d\sigma }\rangle =(-\frac{1}{\pi })\int _{-\infty }^{0}d\omega \text {Im}[\langle \langle d_{\sigma } d_{\sigma }^{\dagger }\rangle \rangle ]$ and $\langle d_{{\bar{\sigma }}}^{\dagger }d_{\sigma }^{\dagger }\rangle =(-\frac{1}{\pi })\int _{-\infty }^{0}d\omega \text {Im}[\langle \langle d_{{\bar{\sigma }}}^{\dagger };d_{\sigma }^{\dagger }\rangle \rangle ]$ are the average occupation and *s*-wave pairing amplitude in the QD, respectively. Both quantities should be numerically computed self-consistently.

Thus, the system Hamiltonian given by Eq. (1) can be rewritten as:

4 $$\begin{aligned} H & =  {} H_{N} + H_{N-QD} + \sum _{\sigma }{\tilde{\varepsilon }}_{d\sigma }d_{\sigma }^{\dagger }d_{\sigma } -({\tilde{\Gamma }}_{S} d_{\uparrow }^{\dagger }d_{\downarrow }^{\dagger } + \text {h.c.}) \nonumber \\ & \quad +  {} H_{TSC}, \end{aligned}$$

where $${\tilde{\varepsilon }}_{d\sigma }=\varepsilon _{d\sigma } + U\langle n_{d \sigma } \rangle $$ and $${\tilde{\Gamma }}_{S} = \Gamma _{S} + U\langle d_{\downarrow } d_{\uparrow } \rangle $$.

It should be noted that other methods can be employed to treat the effects of the Coulomb correlations in N-QD-S systems, as e.g., the slave-boson mean-field approximation (SBMFA)^81,87–89^ in the strong correlated limit $U\rightarrow \infty$, where the doubly-occupied state in the QD is traced out. Hence, the transformation introduced by the SBMFA reduces the problem into a Fermi liquid with renormalized parameters ${\tilde{\Gamma }}_{N,S}$ and ${\tilde{\varepsilon }}_{d}$^81,89^. However, this approach is valid only in the deep Kondo regime $T_{K}\gg \Delta$, where $T_{K}$ is the Kondo temperature^89^, ruling out the possibility of forming a BCS-like singlet in the QD ground state. A faithful analysis of the transition between the Kondo spin-singlet and the BCS-like superconducting singlet is only possible via Numerical Renormalization Group (NRG) technique^66,78,81^, which also allows to study the interplay between the Kondo effect and the MBSs in N-QD-S junctions coupled to Majorana nanowires^60^. The analysis of this interplay, as well as the study of Kondo-BCS singlet transition, goes beyond the scope of the present work, in which we limit ourselves to the consideration of the case away from the Kondo regime^86^.

### Sub-gap Andreev conductance

In a N-QD-S system, the total conductance through a QD is given by the sum of two channels, $G_{N}(V) + G_{A}(V)$, where the first term is the normal electron tunneling conductance and the second one is the Andreev conductance^65,80^. $G_{N}(V)$ gives dominant contribution to the transport outside the gap ($|eV|\ge \Delta$), while $G_{A}(V)$ contributes mainly to the subgap electronic transport ($|eV|< \Delta$). At very low temperatures, when the bias-voltage *eV* applied between the normal and superconducting reservoirs is far from the superconducting gap edges ($|eV|\ll \Delta$), the electronic transport takes place exclusively due to the process of Andreev reflection^90^, see Fig. 1b. The corresponding differential Andreev conductance can be calculated as^58,60,91^:

5 $$\begin{aligned} G_{A}(V)= \frac{2e^{2}}{h}[{\mathcal {T}}_{A}(\omega = -eV) + {\mathcal {T}}_{A}(\omega = eV)], \end{aligned}$$

where $$eV\equiv \mu _{N}-\mu _{S}$$ and

6 $$\begin{aligned} {\mathcal {T}}_{A}(\omega ) = \Gamma _{N}^{2}\sum _{\sigma }[|\langle \langle d_{\sigma }^{\dagger }; d_{{\bar{\sigma }}}^{\dagger } \rangle \rangle |^{2} ] \end{aligned}$$

is the sub-gap transmittance due to Andreev reflection processes, which depends on the anomalous Green’s functions $\langle \langle d_{{\bar{\sigma }}}^{\dagger }; d_{\sigma }^{\dagger } \rangle \rangle$ in the spectral domain $\omega$, with $\Gamma _{N}=\pi \sum _{{\varvec{k}}}|V_{N {\varvec{k}} \sigma }|^{2}\delta (\omega - \varepsilon _{{\varvec{k}}}^{\alpha })$ being the effective broadening of the QD energy levels.

### Green’s functions derivation

In order to get the anomalous Green’s functions related to ${\mathcal {T}}_{A}$, as well as the usual Green’s functions of the QD $\langle \langle d_{\sigma }; d_{\sigma }^{\dagger } \rangle \rangle$, we apply the equation-of-motion technique^84,92^, resulting in the following equation: $\omega \langle \langle A_{i\sigma };B_{j\sigma '}\rangle \rangle =\langle [A_{i\sigma },B_{j\sigma '}]_{+}\rangle +\langle \langle [A_{i\sigma },H];B_{j\sigma '}\rangle \rangle ,$ where $\omega =\omega +\imath \ 0^{+}$ is the spectral frequency, $A_{i\sigma }$ and $B_{j\sigma '}$ are usual fermionic operators belonging to the system Hamiltonian *H* [Eq. (4)]. As we use Hartree–Fock approximation, the system Hamiltonian given by Eq. (4) is bilinear, which allows to close the system of the equations for normal and anomalous Green’s functions, and represent it in the following form:

7 $$\begin{aligned} \hat{{\varvec{G}}}_{\sigma }^{r}(\omega )=\begin{bmatrix}\langle \langle d_{\sigma };d_{\sigma }^{\dagger }\rangle \rangle \\ \langle \langle d_{{\bar{\sigma }}};d_{\sigma }^{\dagger }\rangle \rangle \\ \langle \langle d_{\sigma }^{\dagger };d_{\sigma }^{\dagger }\rangle \rangle \\ \langle \langle d_{{\bar{\sigma }}}^{\dagger };d_{\sigma }^{\dagger }\rangle \rangle \\ \langle \langle f;d_{\sigma }^{\dagger }\rangle \rangle \\ \langle \langle f^{\dagger };d_{\sigma }^{\dagger }\rangle \rangle \end{bmatrix}=\begin{bmatrix}g_{\sigma }^{r}(\omega )^{-1} &{} 0 &{} 0 &{} \sigma {\tilde{\Gamma }}_{S}^{*} &{} \lambda _{-\sigma }^{*} &{} \lambda _{+\sigma }^{*}\\ 0 &{} g_{{\bar{\sigma }}}^{r}(\omega )^{-1} &{} -\sigma {\tilde{\Gamma }}_{S}^{*} &{} 0 &{} \lambda _{-{\bar{\sigma }}}^{*} &{} \lambda _{+{\bar{\sigma }}}^{*}\\ 0 &{} -\sigma {\tilde{\Gamma }}_{S} &{} {\tilde{g}}_{\sigma }^{r}(\omega ) &{} 0 &{} -\lambda _{+\sigma } &{} -\lambda _{-\sigma }\\ \sigma {\tilde{\Gamma }}_{S} &{} 0 &{} 0 &{} {\tilde{g}}_{{\bar{\sigma }}}^{r}(\omega ) &{} -\lambda _{+{\bar{\sigma }}} &{} -\lambda _{-{\bar{\sigma }}}\\ \lambda _{-\sigma } &{} \lambda _{-{\bar{\sigma }}} &{} -\lambda _{+\sigma }^{*} &{} -\lambda _{+{\bar{\sigma }}}^{*} &{} g_{\text {M}}^{r}(\omega )^{-1} &{} 0\\ \lambda _{+\sigma } &{} \lambda _{+{\bar{\sigma }}} &{} -\lambda _{-\sigma }^{*} &{} -\lambda _{-{\bar{\sigma }}}^{*} &{} 0 &{} {\tilde{g}}_{\text {M}}^{r}(\omega )^{-1} \end{bmatrix}^{-1}\cdot \begin{bmatrix}1\\ 0\\ 0\\ 0\\ 0\\ 0 \end{bmatrix}, \end{aligned}$$

where $\lambda _{\pm \sigma }=(\lambda _{L\sigma }\pm \lambda _{R\sigma })/\sqrt{2}$, $g_{\sigma }^{r}(\omega )^{-1}=\omega -{\tilde{\varepsilon }}_{d\sigma }+\imath \Gamma _{\text {N}}$, ${\tilde{g}}_{\sigma }^{r}(\omega )^{-1}=\omega +{\tilde{\varepsilon }}_{d\sigma }+\imath \Gamma _{\text {N}}$, $g_{\text {M}}^{r}(\omega )^{-1}=\omega -\varepsilon _{\text {M}}$, ${\tilde{g}}_{\text {M}}^{r}(\omega )^{-1}=\omega +\varepsilon _{\text {M}}$ and $\sigma {\tilde{\Gamma }}_{S}=-{\tilde{\Gamma }}_{S},+{\tilde{\Gamma }}_{S}$ for $\sigma = \downarrow ,\uparrow$. It is worth mentioning that Eq. (7) has the shape similar to those derived by Zienkiewicz *et al.*^61^, Górski and Kucab^77^ (without the S reservoir) and Ramos–Andrade *et al.*^93^ (for a QD between N leads and side coupled to two TSC nanowires). However, in none of these references finite $\lambda _{R}$, necessary for the estimation of the degree of Majorana nonlocality, was introduced.

## Data Availability

The data that support the findings of this study are available from the corresponding author upon reasonable request.
